# Characterizing and Quenching Autofluorescence in Fixed Mouse Adrenal Cortex Tissue

**DOI:** 10.3390/ijms24043432

**Published:** 2023-02-08

**Authors:** Nawar Sakr, Olga Glazova, Liudmila Shevkova, Nikita Onyanov, Samira Kaziakhmedova, Alena Shilova, Maria V. Vorontsova, Pavel Volchkov

**Affiliations:** 1 Endocrinology Research Centre, Moscow 117292, Russia; 2 Genome Engineering Lab, Moscow Institute of Physics and Technology, Dolgoprudniy 141700, Russia; 3 Faculty of Medicine, M.V. Lomonosov Moscow State University, 27-1, Lomonosovsky Prospect, Moscow 117192, Russia

**Keywords:** autofluorescence, adrenal cortex, fluorescence microscopy, confocal laser scanning microscopy, immunofluorescence

## Abstract

Tissue autofluorescence of fixed tissue sections is a major concern of fluorescence microscopy. The adrenal cortex emits intense intrinsic fluorescence that interferes with signals from fluorescent labels, resulting in poor-quality images and complicating data analysis. We used confocal scanning laser microscopy imaging and lambda scanning to characterize the mouse adrenal cortex autofluorescence. We evaluated the efficacy of tissue treatment methods in reducing the intensity of the observed autofluorescence, such as trypan blue, copper sulfate, ammonia/ethanol, Sudan Black B, TrueVIEW^TM^ Autofluorescence Quenching Kit, MaxBlock^TM^ Autofluorescence Reducing Reagent Kit, and TrueBlack^TM^ Lipofuscin Autofluorescence Quencher. Quantitative analysis demonstrated autofluorescence reduction by 12–95%, depending on the tissue treatment method and excitation wavelength. TrueBlack^TM^ Lipofuscin Autofluorescence Quencher and MaxBlock^TM^ Autofluorescence Reducing Reagent Kit were the most effective treatments, reducing the autofluorescence intensity by 89–93% and 90–95%, respectively. The treatment with TrueBlack^TM^ Lipofuscin Autofluorescence Quencher preserved the specific fluorescence signals and tissue integrity, allowing reliable detection of fluorescent labels in the adrenal cortex tissue. This study demonstrates a feasible, easy-to-perform, and cost-effective method to quench tissue autofluorescence and improve the signal-to-noise ratio in adrenal tissue sections for fluorescence microscopy.

## 1. Introduction

Fluorescence microscopy is an imaging technique that allows the excitation of fluorescent molecules and the detection of the emitted signal over a wide range of wavelengths [1]. Fluorescence microscopy has several advantages over other types of microscopy, including the ability to selectively visualize one or more target molecules in the studied material with high sensitivity and signal-to-noise ratio [2,3]. Advances in fluorescent microscopy have transformed our understanding of biological processes. However, despite the development of numerous new methods for tissue processing and fluorophore imaging, the signal-to-noise ratio remains a recurrent issue in clinical and experimental investigations that use fluorescence microscopy for diagnostic and research applications. One of the primary sources of noise in fluorescence microscopy is autofluorescence (AF). Autofluorescence is the endogenous and exogenous fluorescence that occurs in cells and tissues across a broad range of excitation and emission wavelengths and is unrelated to the specific signal obtained during a fluorescence-based assay [4,5,6]. Exogenous AF results from chemically modified molecules due to tissue processing and fixation procedures [7]. The endogenous AF originates from naturally fluorescent intracellular molecules, such as flavins, flavoproteins, and lipofuscin-like substances [5,8]. Moreover, red blood cells and extracellular tissue components, mainly collagen and elastin, are common causes of AF [5,6,9,10]. AF can obscure or interfere with the signal from labeled cells and tissue sections [10,11], complicating sample examination and interpretation of results, particularly in quantitative studies.

The number of naturally fluorescent molecules varies in different tissue types, making some tissues more autofluorescent than others [12,13]. Adrenal glands are endocrine organs characterized by high lipid content [14,15]. Cells of the adrenal cortex accumulate large amounts of lipid droplets, which serve as storage for cholesterol esters, the precursors of steroid hormones [16,17,18]. Some lipids exhibit AF and complicate the use of fluorescence microscopy in adrenal tissue [19,20]. In addition, the adrenal cortex of various species is rich in autofluorescent pigments. These cortical pigments are typically lipofuscin depositions [15,21,22,23,24]. Lipofuscin, also known as the age pigment, is a yellow-brownish lipid pigment that accumulates in the lysosomes of the cells as they age. The most characteristic feature of lipofuscin is AF. Due to its broad excitation and emission spectra, the lipofuscin AF spectrum overlaps with those of commonly used fluorochromes [4,25,26]. Therefore, the large amounts of fluorescent molecules in adrenal tissue with broad excitation and emission wavelengths are problematic for fluorescence microscopy. Reducing adrenal tissue AF is necessary to distinguish specific labels from AF and improve the signal-to-noise ratio. Several strategies have been applied to diminish AF of fixed tissues, such as photobleaching, chemical treatments, dyes that stain specific tissue components, and combinations of these treatments [27,28,29]. However, AF reduction efficiency differs depending on tissue type and processing method, and, to date, no general formula for quenching AF in various tissue types is currently available [11,29]. A systematic study to analyze AF reduction methods in mouse adrenal cortex tissue has yet to be performed.

In the current study, we characterized the AF in PFA-fixed mouse adrenal cortex tissue sections. We addressed the difficulties of using fluorescent microscopy caused by the observed AF. We compared the effect of several reported treatments for AF reduction on the AF profile of mouse adrenal cortex, such as trypan blue, copper sulfate, ammonia/ethanol, Sudan Black B, TrueVIEW^TM^ Autofluorescence Quenching Kit, MaxBlock^TM^ Autofluorescence Reducing Reagent Kit, and TrueBlack^TM^ Lipofuscin Autofluorescence Quencher. We further evaluated the subsequent tissue immunofluorescence and enhanced green fluorescent protein (EGFP) detection using confocal laser scanning microscopy. Our results show that using TrueBlack^TM^ Lipofuscin Autofluorescence Quencher is the best approach to quench adrenal tissue AF while allowing the detection of target fluorescent labels.

## 2. Results

### 2.1. Evaluation of Autofluorescence in Mouse Adrenal Tissue

**Figure 1:**
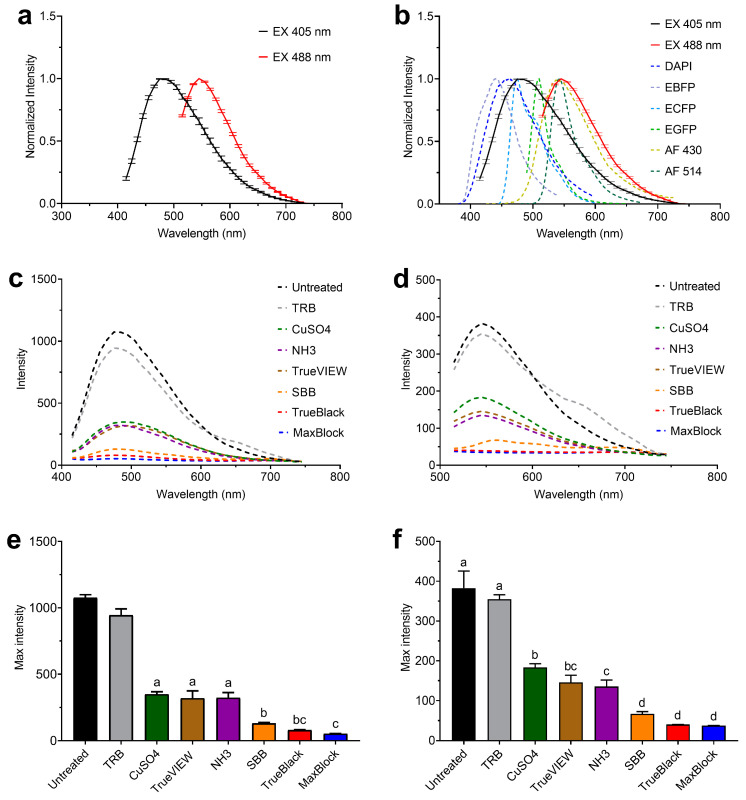
Autofluorescence (AF) emission of untreated PFA-fixed mouse adrenal cortex tissue sections and sections treated with AF reduction methods. Emission line graph of normalized AF emission from untreated adrenal cortex at 405 nm and 488 nm excitation wavelengths (**a**). The interference of adrenal cortex AF spectrum with the spectra of commonly used fluorescent proteins and dyes (**b**). AF emission intensity of untreated adrenal cortex and tissue section treated with AF reduction methods at 405 nm excitation (**c**) and 488 nm excitation (**d**). The means of maximum AF intensity from untreated and treated adrenal cortex sections at 405 nm excitation (**e**) and 488 nm excitation (**f**). ANOVA results are indicated by letters in column superscript. Treatments sharing one letter are not significantly different. Treatments not sharing a letter are significantly different (*p* < 0.05). Bars denote the SD. The AF emission of the adrenal cortex was acquired in the λ-spectral mode using the confocal microscope Olympus FluoView™ FV3000. Spectra of fluorescent proteins and dyes in (**b**) were obtained from the database of fluorescent dyes (www.fluorophores.tugraz.at, accessed on 26 November 2022). Abbreviations: EX, excitation; DAPI, 4′,6-diamidino-2-phenylindole; EBFP, enhanced blue fluorescent protein; ECFP, enhanced cyan fluorescent protein; EGFP, enhanced green fluorescent protein; AF 430, Alexa Fluor 430; AF 514, Alexa Fluor 514; TRB, trypan blue; TrueVIEW, TrueVIEW^TM^ Autofluorescence Quenching Kit; SBB, Sudan Black B; TrueBlack, TrueBlack^TM^ Lipofuscin Autofluorescence Quencher; MaxBlock, MaxBlock^TM^ Autofluorescence Reducing Reagent Kit.

**Figure 2:**
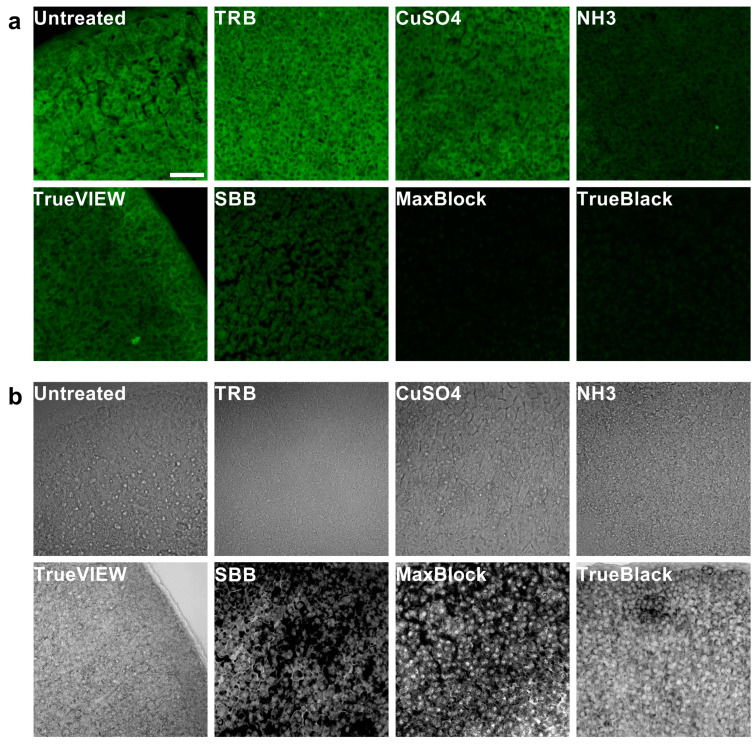
Reducing green wavelength autofluorescence in PFA-fixed mouse adrenal cortex tissue sections using various AF reduction methods. The effect of different tissue treatments on the adrenal cortex green wavelength autofluorescence (**a**), and tissue staining and integrity (**b**). Images were acquired with Olympus FluoView™ FV3000 using 488 nm excitation and a 500–600 nm detection range. Transmitted images were taken simultaneously at 488 nm excitation. Bar: 50 μm. Abbreviations: TRB, trypan blue; TrueVIEW, TrueVIEW^TM^ Autofluorescence Quenching Kit; SBB, Sudan Black B; TrueBlack, TrueBlack^TM^ Lipofuscin Autofluorescence Quencher; MaxBlock, MaxBlock^TM^ Autofluorescence Reducing Reagent Kit.

We analyzed the autofluorescence spectrum of the PFA-fixed mouse adrenal cortex tissue sections using the Olympus FluoView™ FV3000 confocal microscope in lambda scan mode. We collected the emission spectra of the adrenal cortex at 405 nm and 488 nm excitation wavelengths. The normalized emission intensity showed a broad AF emission at 405 nm and 488 nm excitations, with a central emission peak between 475–485 nm and 545–555 nm, respectively (Figure 1a). The intensity of AF was higher at 405 nm excitation compared to 488 nm (Figure 1c,d). Confocal laser scanning microscope (CLSM) images at 488 nm excitation wavelength showed a bright green AF across the adrenal cortex. The observed AF was brighter in the zona fasciculata of the adrenal cortex than in the zona reticularis and adrenal capsule. The AF originated from the cortical cells rather than extracellular tissue components. The intracellular autofluorescent molecules were widespread throughout the cytoplasm and less in the nuclei of the cortical cells (Figure 2a).

To assess if the observed AF might complicate the detection of specific fluorescent labels in the adrenal cortex, we compared the AF spectra at 405 nm and 488 nm excitation with those of commonly used fluorophores obtained from the database of fluorescent dyes (www.fluorophores.tugraz.at, accessed on 26 November 2022). The wide spectrum of AF at 405 nm excitation interferes with the emission of 4′,6-diamidino-2-phenylin-dole (DAPI), enhanced blue fluorescent protein (EBFP), and enhanced cyan fluorescent protein (ECFP), usually excited at 405 nm wavelength (Figure 1b). The AF spectrum at 488 nm excitation showed to interfere with the emission of enhanced green fluorescent protein (EGFP), Alexa fluor 430, and Alexa fluor 514, usually excited at 488 nm wavelength (Figure 1b). Therefore, the broad adrenal cortex AF interferes with detecting and quantifying several fluorescent labels in the blue and green channels of CLSM.

### 2.2. The Effect of Tissue Treatments on Adrenal Cortex Autofluorescence

In order to reduce the AF of the mouse adrenal cortex tissue sections, we tested the efficacy of seven treatments previously described to decrease AF in multiple cells and tissue types (Table 1). The lambda scan showed that all the applied treatments altered the AF profile at both 405 nm and 488 nm excitations. Trypan blue (TRB) treatment decreased the maximum intensity of AF at 405 nm excitation by 12% ± 2% (SE) (Figure 1c,e). At 488 nm excitation, TRB did not reduce AF intensity but shifted the AF emission to longer wavelengths (Figure 1d,f and Figure 2a). Copper(II) sulfate (CuSO_4_), ammonia/ethanol (NH_3_), and TrueVIEW^TM^ Autofluorescence Quenching Kit (TrueVIEW) reduced AF maximum intensity by 68% ± 0.8% (SE), 70% ± 2 (SE), and 70% ± 3% (SE), and by 52% ± 1% (SE), 65% ± 2% (SE), and 62% ± 2% (SE) at 405 nm and 488 nm excitations, respectively. These treatments did not shift the AF emission, and we still observed a central peak of AF emission similar to untreated tissue sections at 405 nm and 488 nm excitations (Figure 1c–f). Figure 2a shows that CuSO_4_, NH_3_, and TrueVIEW reduced the overall background AF. NH_3_ was the most effective among these treatments in reducing the green wavelength AF. However, it did not eliminate the adrenal cortex AF, and autofluorescent granules were still observed after treatment with NH_3_.

Sudan Black B (SBB), TrueBlack^TM^ Lipofuscin Autofluorescence Quencher (TrueBlack), and MaxBlock™ Autofluorescence Reducing Reagent (MaxBlock) further reduced the AF maximum intensity by 88% ± 0.3% (SE), 93% ± 0.1% (SE), and 95% ± 0.03% (SE), and by 82% ± 0.7% (SE), 89% ± 0.04% (SE), and 90% ± 0.07% (SE) at 405 nm and 488 nm excitations, respectively (Figure 1e,f). Emission line graphs showed no central peak of AF emission after treatment with TrueBlack or MaxBlock (Figure 1c,d). SBB reduced AF from the tissue regions showing intense dark staining with SBB. However, AF was still observed in the less stained tissue regions (Figure 2). In contrast, both TrueBlack and MaxBlock reduced the overall AF from the entire adrenal cortex and produced a more homogeneous background. Both treatments mainly stained the cytoplasm of the cortical cells and reduced the cytoplasmic AF to levels lower than the nucleic AF. This staining pattern resulted in slightly brighter nuclei than cytoplasm in the adrenal cortex treated with TrueBlack and MaxBlock (Figure 2a).

### 2.3. Reduction in Autofluorescence from Pigment Accumulations in the Mouse Adrenal Tissue

**Figure 3:**
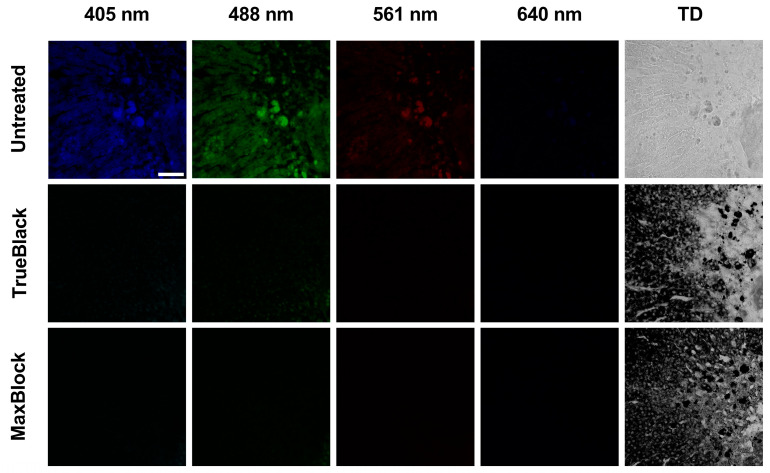
Reduction of autofluorescence (AF) from pigment-laden cells in the adrenal tissue of aged mice. The intense AF of lipofuscin aggregates in the adrenal tissue and the efficacy of TrueBlack and MaxBlock treatment in quenching the AF in the observed channels. Images were taken with Olympus FluoView™ FV3000 using 405, 488, 561, and 640 nm excitation and 430–470, 500–540, 570–620, and 650–750 nm detection ranges, respectively. Transmitted images were taken simultaneously at 640 nm excitation. Bar: 50 μm. Abbreviations: TrueBlack, TrueBlack^TM^ Lipofuscin Autofluorescence Quencher; MaxBlock, MaxBlock^TM^ Autofluorescence Reducing Reagent Kit; TD, transmitted detector.

The autofluorescent lipofuscin accumulates in the adrenal tissue as mice age. The presence of pigment-laden cells containing large amounts of autofluorescent lipoid pigments further complicates the elimination of AF from aged mice’s adrenal tissue. We examined the AF of adrenal tissue sections from aged mice. In addition to the high intracellular AF, we observed several irregularly shaped granules with high-density fluorescence across all observed channels. These granules varied in size and scattered in the adrenal cortex and the corticomedullary junction (Figure 3). We treated the adrenal tissue sections from aged mice with TrueBlack or MaxBlock, which showed the highest efficacy in decreasing AF of the adrenal cortex from younger mice (Figure 1c–f and Figure 2a). CLSM images showed that both treatments quenched the fluorescence from the autofluorescent accumulations. The reduction of this intense AF did not require increased incubation time or working concentration. We noticed a more intense dark staining of these granules and the cortical cells compared to the cells from younger mice (Figure 2b). TrueBlack and MaxBlock specifically stained the cortical cells’ cytoplasm and, to a lesser extent, the nuclei and adrenal medullary cells (Figure 3), similar to findings from younger mice’s adrenal tissue sections (Figure 2b).

### 2.4. The Effect of TrueBlack and MaxBlock Treatments on the Detection of Fluorescent Labels in the Mouse Adrenal Cortex

AF is problematic for fluorescence-based assay in tissue sections. Intrinsic fluorescence interferes with or even masks the specific signals from fluorescent labels. Reducing tissue AF without affecting fluorescent tags is necessary to obtain valid data. To evaluate the applicability of AF treatments in immunofluorescence (IF), we treated adrenal tissue sections with TrueBlack or MaxBlock before (pre-treatment) or after applying the antibodies (post-treatment) for indirect IF. We immunostained the 21-hydroxylase typically expressed in the mouse adrenal cortex and used secondary antibodies conjugated to Alexa Fluor 594 to visualize the 21-hydroxylase staining. We detected the fluorescence signals in the 570–670 nm range at 561 nm excitation and used the 500–540 nm detection range at 488 nm excitation to evaluate the efficacy and stability of AF reduction treatments throughout the immunostaining procedure.

**Figure 4:**
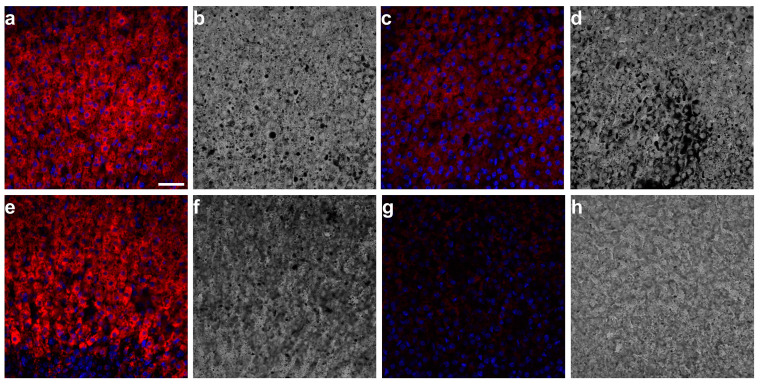
Immunofluorescence staining of fixed adrenal cortex tissue sections treated with TrueBlack or MaxBlock. Mouse adrenal tissue sections stained with DAPI, rabbit anti-CYP21A2 primary antibody, and goat anti-rabbit IgG H&L (Alexa Fluor^TM^ 594) secondary antibody. CYP21A1 staining in tissue sections treated with TrueBlack before (**a**) or after immunostaining (**c**) and the corresponding transmitted images (**b** and **d**, respectively). CYP21A1 staining in tissue sections treated with MaxBlock before (**e**) or after immunostaining (**g**) and the corresponding transmitted images (**f** and **h**, respectively). Images were taken with Olympus FluoView™ FV3000 using 405, 488, and 561 nm excitation and 430–470, 500–540, and 570–670 nm detection ranges, respectively. Fluorescence images (**a**,**c**,**e**,**g**) show the overlay of the three observed channels. Transmitted images were taken simultaneously at 561 nm excitation. Bar: 30 μm. Abbreviations: DAPI, 4′,6-diamidino-2-phenylindole; TrueBlack, TrueBlack^TM^ Lipofuscin Autofluorescence Quencher; MaxBlock, MaxBlock^TM^ Autofluorescence Reducing Reagent Kit.

CLSM images obtained using the same acquisition settings showed that Alexa Flour 594 signals were detectable in adrenal tissue sections pre-treated with TrueBlack or MaxBlock (Figure 4a,e). In contrast, the post-treatment of immunostained sections with either TrueBlack or MaxBlock masked most of the fluorescence signals from the conjugated antibodies. Post-treatment with MaxBlock had the most negative effect on the IF in the adrenal cortex. (Figure 4c,g). We stained tissue sections with DAPI after AF treatments. The treatment of adrenal tissue with TrueBlack or MaxBlock did not mask the fluorescent signals from DAPI in the adrenal cortex. However, DAPI fluorescence was slightly brighter in sections treated with TrueBlack than those treated with MaxBlock (Figure 4a,c,e,g). These findings suggest that in comparison to MaxBlock, TrueBlack treatment has a less adverse effect on the specific fluorescence in IF. Nevertheless, both treatments quenched the adrenal cortex AF at 488 nm excitation when applied before or after immunostaining.

**Figure 5:**
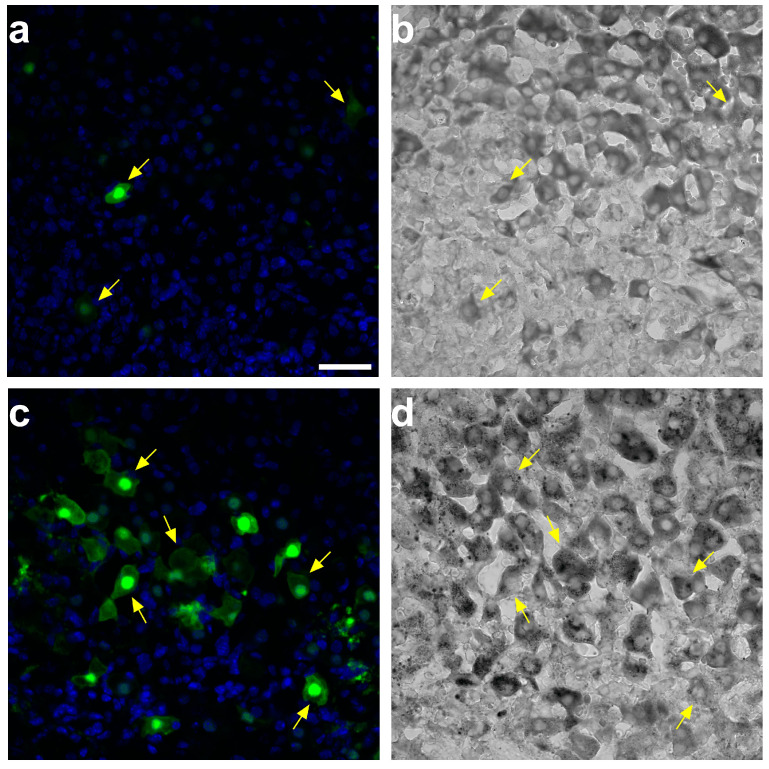
EGFP detection in fixed frozen adrenal tissue sections treated with TrueBlack. The native fluorescence of EGFP in the adrenal tissue treated with TrueBlack (**a**) and the corresponding transmitted image (**b**). EGFP staining with chicken anti-GFP Polyclonal antibody and goat anti-chicken IgY H&L (Alexa Fluor^TM^ 488) after treatment with TrueBlack (**c**) and the corresponding transmitted image (**d**). Arrows represent cells stained with TrueBlack and exhibit green fluorescence. Images in (**a**) and (**b**) were obtained using different acquisition settings. Bar: 30 μm. Abbreviations: TrueBlack, TrueBlack^TM^ Lipofuscin Autofluorescence Quencher.

Lastly, we evaluated the effect of TrueBlack treatment on the native fluorescence of enhanced green fluorescent protein (EGFP). We applied TrueBlack to PFA-fixed frozen adrenal tissue sections from mice injected with recombinant adeno-associated virus vectors carrying the EGFP gene (rAAV-EGFP). The treatment of tissue sections with TrueBlack did not quench the fluorescence of EGFP. We detected EGFP native fluorescence in a number of cells stained with TrueBlack (Figure 5a,b). We enhanced the EGFP fluorescence by indirect immunostaining after TrueBlack treatment and applied secondary antibodies conjugated to Alexa Fluor 488. We detected the fluorescence of stained EGFP from various cells stained with TrueBlack. In most cells, the fluorescence intensity was higher in the cells’ nuclei than in the cytoplasm (Figure 5c,d). Altogether, these results suggest that treatment with TrueBlack eliminates AF of the adrenal cortex while having a minimal effect on the fluorescence of fluorophore-conjugated antibodies and EGFP.

## 3. Discussion

In this study, we demonstrated that the mouse adrenal cortex emits intense AF in the commonly used channels in CLSM. Adrenal tissue AF was widespread in the cortical cells and had broad excitation and emission spectra. The observed AF spectrum interferes with the spectra of many fluorescent proteins and dyes commonly used in fluorescence microscopy (Figure 1a–d and Figure 2a), complicating the detection of fluorescent labels and possibly leading to false positive results. The adrenal cortex AF was brighter at 405 nm and 488 nm excitations compared to longer excitation wavelengths, as shown in Figure 3. Therefore, we used 405 nm and 488 nm excitations to evaluate the efficiency of different tissue treatments against adrenal cortex AF. Several previously described AF-reducing agents decreased the AF of the adrenal cortex. MaxBlock and TrueBlack were the most effective treatments for quenching the intracellular AF at different excitation wavelengths. Other treatment methods, including SBB, NH_3_, CuSO_4_, and TrueVIEW, reduced the AF to a certain extent but did not eliminate the AF of the adrenal cortex (Figure 1e,f and Figure 2a).

Moreover, TrueBlack and MaxBlock treatments quenched AF of pigment accumulations, exhibiting bright AF across multiple CLSM channels (Figure 3). The pre-treatment of adrenal tissue sections with TrueBlack for IF masked the intrinsic AF but not the specific fluorescent signals from the immunolabels (Figure 4a). Similarly, treatment with TrueBlack did not interfere with detecting EGFP in adrenal tissue sections (Figure 5a,c).

The adrenal cortex AF originated mainly from the cortical cells’ cytoplasm (Figure 2a). Cortical cells are known to accumulate lipid droplets containing cholesterol esters for steroid biosynthesis [14,16,17,18]. Some lipid compounds exhibit AF depending on their molecular properties [30]. In addition, the adrenal cortices are rich with intracytoplasmic lipofuscin, which builds up in the lysosomes of cells as they age [15,24]. AF is a distinctive feature and is regarded as a reliable marker of lipofuscin [31,32,33]. Lipofuscin fluorescence emission spectra show considerable heterogeneity due to differences in chemical composition [34]. Hence, the lipofuscin and high lipid content are possible causes of the broad intracellular AF observed in the mouse adrenal cortex. In addition to the endogenous AF sources, fixation with paraformaldehyde can contribute to the AF observed in the adrenal cortex. Crosslinking fixatives such as formaldehyde and glutaraldehyde are major sources of exogenous AF [6,10,27]. During tissue fixation, aldehydes react with the amine groups of proteins and amino acids to form fluorescent complexes known as Schiff bases [7,35].

The reduction in adrenal cortex AF with TrueBlack^TM^ Lipofuscin Autofluorescence Quencher (TrueBlack) and SBB further suggests that lipofuscin and lipids are potential AF sources. TrueBlack is a commercial AF quenching reagent that reduces tissue sections AF from lipofuscin and less efficiently from other sources like red blood cells and extracellular components. TrueBlack has been used for AF reduction in a wide range of human and mouse tissue types, such as the brain [36,37,38,39], retina [40,41], heart [42,43], lung [44,45], and liver tissue [46,47]. SBB is a superlipophilic diazo dye used for staining a wide variety of lipids [48,49] and some proteins [50]. SBB shows a high affinity to lipofuscin in frozen and paraffin-embedded tissue sections [51,52]. Therefore, it has been used to reduce AF from lipids, lipofuscin, and lipofuscin-like substances in various tissue types [4,6,11,13,27,29,53,54,55]. The proposed mechanism is that SBB masks the autofluorescent structures without chemically interacting with the components of these structures [4,29,53]. Similar to TrueBlack and SBB, MaxBlock^TM^ Autofluorescence Reducing Kit (MaxBlock) primarily stained the cytoplasm of cortical cells (Figure 2b), quenching the AF of the adrenal cortex. MaxBlock is a commercialized AF treatment designed to reduce background AF on paraffin-embedded and frozen tissue sections. It has been used to diminish AF in several tissues, such as the liver [56], heart [57], lung [58,59], pancreas [60], and skin [61] tissues.

Although SBB is a more cost-effective AF treatment than TrueBlack and MaxBlock, SBB preparation is laborious and requires longer incubation time to reduce AF of the adrenal cortex. TrueBlack and MaxBlock are ready-to-use reagents that showed more efficiency in reducing AF of the adrenal cortex, resulting in a more homogeneous nonfluorescent background (Figure 1c–f and Figure 2a). Moreover, SBB introduces some AF in the red and far-red channels. SBB is incompatible with antifading agents that preserve the fluorescent labels for long-term storage and analysis [53] and may have adverse effects on the fluorescence of specific labels in tissue IF [4,27].

The commercial TrueVIEW Autofluorescence Quenching Kit (TrueVIEW) reduces tissue AF via treatment with an aqueous solution of nonfluorescent, hydrophilic molecules. These negatively charged molecules bind electrostatically and diminish AF from non-lipofuscin sources such as red blood cells, collagen, elastin, and aldehyde fixation [62]. TrueVIEW has been used to reduce AF in various tissues [63,64,65,66]. TrueView reduced the AF of adrenal cortex cells (Figure 1c–f and Figure 2a), suggesting that lipofuscin and other hydrophobic molecules are not the only sources of AF in the adrenal cortex.

Copper(II) sulfate (CuSO_4_) was reported to reduce tissue AF from lipofuscin [4,67], red blood cells [68,69], and other sources [28,69,70,71]. The chemical mechanism of Cu^2+^ quenching of AF is not precise. It is suggested that Cu^2+^ acts as an electron scavenger that receives electrons from the autofluorescent molecules by collisional contact and circumvent the fluorescence emission [72]. In our study, CuSO_4_ treatment did not eliminate the adrenal cortex AF (Figure 2a), similar to previous reports in some tissue types [27,73]. CuSO_4_ may also have a negative effect on IF signals when used at high concentrations [4,27]. However, we did not investigate the impact of this treatment on IF in adrenal tissue.

NH_3_ reduces tissue AF by dissolving negatively charged lipid derivatives and phenols, hydrolyzing weak esters, and deactivating pH-sensitive fluorochromes [6,29]. In previous reports, NH_3_ reduced AF in bone marrow, kidney, and placenta tissue sections [6,13,28]. Meanwhile, it failed to quench AF in the brain [29], liver, and pancreas tissue [13]. NH_3_ was ineffective against the AF of lipofuscin granules in the myocardial tissue [6]. In our study, NH_3_ reduced the general background AF but did not eliminate the AF of fluorescent granules in the adrenal cortex (Figure 1c–f and Figure 2a).

Trypan Blue (TRB) diffuses into permeabilized cells and distributes uniformly in the cell nucleus and cytoplasm. If used in optimized concentration, TRB reduces AF when dye molecules are at a proper orientation and distance to autofluorescent molecules or when bound to autofluorescent molecules [74]. Contrary to previous reports [28,74,75], TRB did not diminish the AF of the adrenal cortex (Figure 1c–f and Figure 2a), possibly due to the use of a suboptimal concentration or the omission of permeabilization step in order to process all the tissue sections equally before applying the AF treatments. However, consistent with the previous reports [74,75], TRB shifted the AF spectrum of the adrenal cortex to longer wavelengths. We did not further optimize the TRB treatment protocol as the induced AF in the longer wavelengths makes TRB a less suitable option than other tested treatments.

The lipofuscin accumulates in the lysosomes of aged cells because it cannot be eliminated by degradation or exocytosis [76]. Adrenal cortices of aged mice and rats accumulate large amounts of lipofuscin [15,24]. Excessive lipofuscin accumulations can result from the degeneration of cortical cells, dietary and steroid imbalances, and administration of some exogenous chemicals [14]. In addition, mouse adrenal tissue may contain pigment-laden cells. These pigmented cells are usually scattered in the adrenal cortex or at the corticomedullary junction. They are thought to be a consequence of the regression of the transient cortical X-zone [14,77]. The pigment-laden cells cluster as the mouse ages and can coalesce to form multinucleated giant cells. The pigmented cells from animals of different ages show similar histochemical staining properties and exhibit orange-yellow primary fluorescence [78]. Thus, the presence of these highly autofluorescent aggregates with various sizes, pigment concentrations, and localizations decreases the signal-to-noise ratio and complicates the interpretation of fluorescence microscopy results. 

We tested whether TrueBlack or MaxBlock, which eliminated the AF from the smaller intracellular autofluorescent structures, may reduce the AF from pigment accumulations. Both TrueBlack and MaxBlock quenched the AF from the bright autofluorescent accumulations in the adrenal cortex and in the corticomedullary junction (Figure 3). The adrenal cortex and the pigment accumulations showed more intense cytoplasmic staining with both TrueBlack and MaxBlock than in younger mice (Figure 2b). The staining intensity is possibly proportional to intracellular lipofuscin content, which increases as the mice age. These findings highlight the effectiveness of both TrueBlack and MaxBlock in quenching the AF from dense AF aggregates in the mouse adrenal glands of different ages that might be challenging to eliminate using other AF treatments.

The major limitation of tissue AF reduction treatments is their effect on the specific fluorophores used to visualize target molecules. Several AF quenching methods may quench assay-specific signals [4,27,28,74]. TrueBlack and MaxBlock have been used to treat AF in various tissue types. However, in most tissues, AF originates from specific intracellular or extracellular tissue components that have a defined localization within the tissue, and masking this AF with non-fluorescent dyes does not affect the IF signals from other parts of the tissue. In contrast, autofluorescent molecules are widespread across the adrenal cortex, with high density in the cytoplasm of most cells. This wide distribution causes intense dark staining across the adrenal cortex after treatment with AF-reducing dyes (Figure 2b). 

We investigated the effect of the staining with TrueBlack and MaxBlock on IF signals from the cortical cells. The pre-treatment of adrenal tissue sections with TrueBlack before IF had a mild effect on the fluorescence of conjugated antibodies (Figure 4a). Conversely, post-treatment with MaxBlock had the most adverse impact on IF signals (Figure 4g). We also tested if TrueBlack treatment interferes with the fluorescence of enhanced green fluorescence protein (EGFP). EGFP is a versatile biological marker for visualizing protein localization, monitoring transgenic expression, and tracking specific cell types in the adrenal cortex. The broad excitation and emission of AF in the adrenal cortex interfere with detecting EGFP (Figure 1b). We visualized the fluorescence of both native and immunolabeled EGFP in cortical cells stained with TrueBlack in fixed frozen adrenal tissue sections (Figure 5). These findings suggest that TrueBlack treatment is compatible with IF and EGFP fluorescence in adrenal tissue sections, as it diminishes tissue AF without masking the fluorescent signals from fluorescent labels.

Lastly, it is worth noting that the signal of fluorescent labels from treated adrenal cortical cells may be inversely related to the staining intensity of these cells with AF-reducing dyes. The concentration of the dye for AF reduction, incubation time, and tissue content of autofluorescent materials possibly determine the staining intensity of treated tissue sections and, hence, the interference with the detection of specific fluorophores. A significant advantage of TrueBlack over other ready-to-use reagents is the ability to optimize both working concentration and incubation time. TrueBlack is provided as a 20X solution that can be diluted in ethanol according to the required dilution, usually 1X, according to the manufacturer’s instructions. However, TrueBlack has been used in different concentrations [10,79,80] and incubation times [41,81,82] for AF reduction. The ability to optimize TrueBlack treatment is helpful when treating tissues rich with autofluorescent material, such as the adrenal glands, to avoid IF signal masking by intensive dark tissue staining. As mentioned before, the lipofuscin levels in the adrenal cortex can vary with age, diet, hormonal imbalances, and other factors. We recommend testing different concentrations and incubation times with TrueBlack to reach the best balance between tissue AF reduction and target fluorophore visualization and to achieve the required signal-to-noise ratio.

## 4. Materials and Methods

### 4.1. Animals

The animals were housed and utilized for experimental procedures in compliance with Directive 2010/63/EU and the recommendations of the local Bioethical Committee of Lomonosov Moscow State University.

### 4.2. Tissue Sections

Eight week old C57BL/6J mice were anesthetized and sacrificed under full anesthesia with isoflurane (Miralek, Moscow, Russia). Adrenal glands were dissected and immersed in 4% PFA (PanReac AppliChem ITW Reagents, Barcelona, Spain) in PBS for 16 h at 4 °C [83]. The adrenal glands were washed three times with PBS for 15 min each, transferred to a solution of 15% sucrose in PBS for 24 h at 4 °C, and then moved to a solution of 30% sucrose in PBS for 48 h at 4 °C. The adrenal glands were then embedded and frozen in Tissue-Tek^TM^ O.C.T. Compound (Sakura Finetek, Warsaw, Poland). Sections of 12 μm were cut and mounted onto Polysine adhesion slides (Thermo Fisher Scientific, Waltham, MA, USA). Tissue sections were stored in the dark at −20 °C for subsequent use. 

Archival 4% PFA-fixed frozen tissue sections from aged mice and mice injected with recombinant AAV vector carrying the gene of enhanced green fluorescent protein (rAAV-EGFP) were previously prepared similarly and stored in the dark at −20 °C till usage.

### 4.3. Tissue Treatment for Reducing Autofluorescence

Frozen mouse adrenal tissue sections were thawed for 30 min at RT and washed with PBS two times, 10 min each, to remove O.C.T. The tissue sections were then treated separately with tissue AF treatment methods (Table 1) at room temperature. Sudan Black B (SBB, Dia-m, Moscow, Russia) was prepared as 0.1% (W/V) in 70% ethanol, as described previously [27]. Sections were incubated with the SBB solution sealed airtight in the dark for 20 min, and then dipped briefly in 70% ethanol once before washing with PBS. A solution of 10 mM copper(II) sulfate (CuSO_4_) in 50 mM ammonia acetate, pH 5, was prepared and applied to sections for 90 min [27]. Ammonia/ethanol (NH_3_) was prepared as 0.25% (V/V) ammonia (PanReac AppliChem ITW Reagents, Barcelona, Spain) in 70% ethanol and applied to tissue sections for 1 h [29]. A fresh 0.05% (W/V) trypan blue (Paneko, Moscow, Russia) in PBS solution was prepared and applied to slides for 15 min [28]. The 20X TrueBlack solution (TrueBlack^TM^ Lipofuscin Autofluorescence Quencher, Biotium, Fremont, CA, USA) was diluted to 1X in 70% ethanol and applied to tissue sections for 1 min. After each of these treatments, the slides were washed with PBS 3 times for 15 min each. TrueVIEW Reagent (TrueVIEW^TM^ Autofluorescence Quenching Kit, Vector Laboratories, Burlingame, CA, USA) was prepared according to manufacturer instructions, applied immediately to sections for 3 min, and then washed once with PBS for 5 min. MaxBlock^TM^ Autofluorescence Reducing Reagent Kit (MaxVision Biosciences, Bothell, WA, USA) was applied according to manufacturer instructions. Tissue sections were incubated with MaxBlock^TM^ Autofluorescence Reducing Reagent (Reagent A) for 1 min, washed according to manufacturer instructions, incubated with Post-Detection Conditioner (Reagent B) for 5 min, and then washed according to manufacturer instructions. After each treatment, the slides were mounted onto coverslips with a polyvinyl alcohol mounting medium with DABCO^TM^ antifading (Sigma-Aldrich, St. Louis, MO, USA). The slides treated with TrueVIEW^TM^ Autofluorescence Quenching Kit were mounted with VECTASHIELD Vibrance Antifade Mounting Medium (Vector Laboratories, Burlingame, CA, USA) according to the manufacturer’s instructions. The slides were stored at 4 °C in the dark for subsequent examination.

### 4.4. Autofluorescence Emission Spectra and Images Acquisition

For each treatment and control group, the AF emission spectra were acquired in a λ-spectral mode from the adrenal cortex tissue sections (*n* = 4) using the confocal microscope Olympus FluoView™ FV3000 (Olympus, Tokyo, Japan) with a UPLXAPO40X, 0.95 NA dry objective (Olympus) using the following settings: OneWay capture; 512 × 512 pixel format; Airy disk, 1 AU; line averaging, 2. Tissue sections were excited with a 405 nm laser diode (OBIS 405 nm LX 50 mW, Coherent, Singapore) and a 488 nm laser diode (OBIS 488 nm LS 20 mW, Coherent, Singapore) at 100% laser power while using the excitation dichroic mirror (ExDM) BS10/90. ExDM BS10/90 allows approximately 10% of the power of the selected laser to pass through the mirror, and 90% of the emitted light back through. Emission data were collected using the gallium arsenide phosphide (GaAsP) photomultiplier tube (PMT) high-sensitivity spectral detector. The PMT settings were as follows: detector voltage, 650 V; gain, 1; offset, 3. We used detection ranges of 415–745 nm and 515–745 nm at 405 nm and 488 nm excitations, respectively. The detection bandwidth and the detection step size were 10 nm. The Olympus FluoView^TM^ FV3000 ‘Series analysis’ tool was used to analyze the emission data [84], and the average intensity values were exported for further analysis. Images demonstrating AF levels in the green wavelengths and transmitted images were acquired at excitation of 488 nm and detection range 500–600 nm, with a UPLXAPO40X, 0.95 NA Dry objective using the following settings: OneWay capture; 2048 × 2048 pixel format; Airy disk, 1 AU; line averaging, 5. The images were exported without enhancements or manipulations.

### 4.5. Immunofluorescence

Adrenal tissue sections were thawed at RT for 30 min and washed with PBS two times, 10 min each, to remove O.C.T. After that, the sections were permeabilized with PBS containing 0.1% Triton X-100 (AppliChem GmbH, Darmstadt, Germany) for 10 min, and then blocked with PBS containing 10% goat serum (Abcam, Cambridge, UK) for 2 h at RT in a humidified chamber. The tissue sections were incubated with the primary antibodies diluted in PBS containing 1% BSA (Dia-m, Moscow, Russia), 0.25% Triton X-100, and 0.25% Tween-20 (Bio-Rad, Hercules, CA, USA) for 16 h at 4 °C. After incubation, the sections were washed twice with PBS containing 0.1% Triton X-100 and Tween-20 for 10 min each. The tissue sections were incubated with the secondary antibodies diluted in PBS containing 1% BSA, 0.25% Triton X-100, and 0.25% Tween-20 for 2 h at RT in a humidified chamber, and then washed two times with PBS containing 0.1% Triton X-100 and Tween-20, 10 min each.

Tissue sections were stained with 0.5 µg/mL 4′,6-diamidino-2-phenylindole (DAPI) for 10 min, washed with PBS for 10 min, mounted onto coverslips with polyvinyl alcohol mounting medium with DABCO^TM^ antifading, dried overnight, and stored in the dark at 4 °C until examination. Images of IF were acquired with the confocal microscope Olympus FluoView™ FV3000 UPLXAPO60XO, 1.42 NA Oil Immersion Objective.

TrueBlack and MaxBlock AF reduction treatments were applied either after the blocking step (pre-treatment) or after the incubation with secondary antibodies (post-treatment). For pre-treated sections, Triton X-100 and Tween-20 were excluded from all the solutions after applying TrueBlack or MaxBlock without changing the buffers’ other ingredients.

The primary antibodies used for IF were Anti-CYP21A2 antibody (1:500, ab232701, Abcam) and GFP polyclonal antibody (1:500, A10262, Invitrogen, Eugene, OR, USA). The secondary antibodies used for IF were goat anti-rabbit IgG H&L (Alexa Fluor^TM^ 594) (1:1000, ab150080, Abcam) and goat anti-chicken IgY H&L (Alexa Fluor^TM^ 488) (1:1000, ab150169, Abcam).

### 4.6. Data Analysis

The average intensity values at 405 nm and 488 nm in λ-spectral mode were imported into GraphPad Prism software version 8.0.1. (GraphPad Software, Inc., San Diego, CA, USA) In order to examine the adrenal cortex AF spectral shape, the emission data for untreated control were normalized, and the mean normalized intensity and the standard deviation were plotted. The mean normalized intensities of untreated control at 405 nm and 488 nm excitation were compared with emission spectra of fluorescent proteins and synthetic fluorescent dyes publicly available in the database of fluorescent dyes (www.fluorophores.tugraz.at, accessed on 26 November 2022) to assess the degree of AF interference with the commonly used fluorophores in fluorescence microscopy. After each AF treatment, the means of average emission intensity values were plotted at 405 nm and 488 nm excitations and visually examined for differences in emission intensity and spectrum shape. 

In addition, the mean, standard deviation, and standard error for the maximum emission intensities at 405 nm and 488 nm excitations were calculated for each AF treatment. Maximum intensities were compared using one-way ANOVA with Tukey’s multiple comparisons test for pairwise comparisons of treatments. Statistical significance was indicated by letters in superscript. Treatments that share the same letter are not different from each other, while treatments not sharing a letter are significantly different. The percentage difference of maximum intensity at 405 nm and 488 nm excitation between each treatment and the untreated control with the standard error of the mean was calculated to evaluate each treatment’s efficiency in reducing mouse adrenal cortex tissue AF.

## 5. Conclusions

In this study, we assessed the characteristics of adrenal cortex tissue AF and examined several treatments for diminishing the observed AF. We found TrueBlack to be efficacious in quenching AF in the mouse adrenal cortex while preserving the signals of specific fluorescent labels. This study provides a practical method for identifying and eliminating AF during fluorescence-based assays in mouse adrenal tissue sections.

## Figures and Tables

**Table 1:** Summary of Autofluorescence Treatments.

Treatment	Abbreviation or Chemical Formula
Trypan blue	TRB
Copper(II) sulfate	CuSO_4_
Ammonia/ethanol	NH_3_
Sudan Black B	SBB
TrueVIEW^TM^ Autofluorescence Quenching Kit	TrueVIEW
MaxBlock™ Autofluorescence Reducing Reagent Kit	MaxBlock
TrueBlack^TM^ Lipofuscin Autofluorescence Quencher	TrueBlack

## Data Availability

Not applicable.
